# Supplementary material for: Implementing and evaluating far-field 3D X-ray diffraction at the I12 JEEP beamline, Diamond Light Source
Source: J Synchrotron Radiat. 2022 May 16;29(Pt 4):1043–53. doi: 10.1107/S1600577522004088 (PMC9255569; doi:10.1107/S1600577522004088)
Supplement: Supplementary file 1 [file s-29-01043-sup1.pdf]

JOURNAL OF SYNCHROTRON RADIATION

Volume 29 (2022)

Supporting information for article:

Implementing and evaluating far-field 3D X-ray diffraction at the I12 JEEP beamline, Diamond Light Source

James A. D. Ball, Anna Kareer, Oxana V. Magdysyuk, Stefan Michalik, Anastasia Vrettou, Neal Parkes, Thomas Connolley and David M. Collins

Implementing and Evaluating Far-Field 3D X-Ray Diffraction at the I12 JEEP Beamline, Diamond Light Source

James A. D. Ball[a,b], Anna Kareer[c], Oxana V. Magdysyuk[b], Stefan Michalik[b], Anastasia Vrettou[a], Neal Parkes[a], Thomas Connolley[b], and David M. Collins *[a]

[a]School of Metallurgy and Materials, University of Birmingham, Edgbaston, Birmingham B15 2TT, United Kingdom
[b]Diamond Light Source Ltd., Harwell Science and Innovation Campus, Didcot OX11 0DE, United Kingdom
[c]Department of Materials, University of Oxford, Oxford, OX1 3PH, United Kingdom

Supplementary Content

The table below lists each software package that was used in the data analysis of 3DXRD data collected from the I12 beamline, Diamond Light Source.

Table 1: Software package usage in data analysis process.

**Software package**	**Reference**	**Usage**
ImageD11	Wright (2020)	Peak searching, merging, cleaning
xfab	Sørensen *et al.* (2021)	Orientation error determination
GrainSpotter	Schmidt (2014)	Initial index
FitAllB	Oddershede *et al.* (2010)	Refinement of lattice parameter and strain
numpy	Harris *et al.* (2020)	All stages
pandas	McKinney (2010)	Internal database management
matplotlib	Hunter (2007)	Graph plotting
pymicro	Proudhon (2021)	Grain tracking
scipy	Virtanen *et al.* (2020)	Letterbox stitching
h5py	Collette *et al.* (2021)	Detector image processing
Pillow	Van Kemenade *et al.* (2021)	Detector image processing
fabio	Knudsen *et al.* (2013)	Detector image processing
jsmin	de Jager (2021)	Processing input files
MTEX	Bachmann *et al.* (2010)	Grain volume distribution

*d.m.collins@bham.ac.uk

References

Bachmann, F., Hielscher, R. and Schaeben, H. (2010). *Solid State Phenomena*, **160**, 63–68.
URL: *https://www.scientific.net/SSP.160.63*

Collette, A., Kluyver, T., Caswell, T. A., Tocknell, J., Kieffer, J., Scopatz, A., Jelenak, A., Dale, D., Chen, Payno, Juliagarriga, Vincent, T., Sciarelli, P., Valls, V., Ghosh, S., Pedersen, U. K., Jakirkham, Raspaud, M., Danilevski, C., Abbasi, H., Readey, J., Paramonov, A., Chan, L., Solé, V. A., Jialin, Feng, Y., Vaillant, G. A., Teichmann, M., Brucher, M. and Johnson, S. R., (2021). H5py/h5py: 3.3.0. Zenodo.
URL: *https://zenodo.org/record/5012627*

Harris, C. R., Millman, K. J., van der Walt, S. J., Gommers, R., Virtanen, P., Cournapeau, D., Wieser, E., Taylor, J., Berg, S., Smith, N. J., Kern, R., Picus, M., Hoyer, S., van Kerkwijk, M. H., Brett, M., Haldane, A., del Río, J. F., Wiebe, M., Peterson, P., Gérard-Marchant, P., Sheppard, K., Reddy, T., Weckesser, W., Abbasi, H., Gohlke, C. and Oliphant, T. E. (2020). *Nature*, **585**(7825), 357–362.
URL: *https://www.nature.com/articles/s41586-020-2649-2*

Hunter, J. D. (2007). *Computing in Science Engineering*, **9**(3), 90–95.

de Jager, T., (2021). Jsmin - JavaScript minifier.
URL: *https://github.com/tikitu/jsmin*

Knudsen, E. B., Sørensen, H. O., Wright, J. P., Goret, G. and Kieffer, J. (2013). *Journal of Applied Crystallography*, **46**(2), 537–539.
URL: *http://scripts.iucr.org/cgi-bin/paper?kk5124*

McKinney, W. (2010). In *Proceedings of the 9th Python in Science Conference*, pp. 56–61.
URL: *http://conference.scipy.org/proceedings/scipy2010/mckinney.html*

Oddershede, J., Schmidt, S., Poulsen, H. F., Sørensen, H. O., Wright, J. and Reimers, W. (2010). *Journal of Applied Crystallography*, **43**(3), 539–549.
URL: *http://scripts.iucr.org/cgi-bin/paper?ks5240*

Proudhon, H., (2021). Pymicro.
URL: *https://github.com/heprom/pymicro*

Schmidt, S. (2014). *Journal of Applied Crystallography*, **47**(1), 276–284.
URL: *http://scripts.iucr.org/cgi-bin/paper?rg5034*

Sørensen, H., Oddershede, J. and Wright, J., (2021). FABLE-3DXRD/xfab.
URL: *https://github.com/FABLE-3DXRD/xfab*

Van Kemenade, H., Murray, A., Wiredfool, Clark, A., Karpinsky, A., Baranovič, O., Gohlke, C., Dufresne, J., Crowell, B., Schmidt, D., Kopachev, K., Houghton, A., Mani, S., Landey, S., Vashek, Ware, J., Douglas, J., Stanislau, T., Caro, D., Martinez, U., Kossouho, S., Lahd, R., Lee, A., Brown, E. W., Tonnhofer, O., Bonfill, M., Rowlands, P., Al-Saidi, F. and Novikov, G., (2021). Python-pillow/Pillow:. Zenodo.
URL: *https://zenodo.org/record/5076624*

Virtanen, P., Gommers, R., Oliphant, T. E., Haberland, M., Reddy, T., Cournapeau, D., Burovski, E., Peterson, P., Weckesser, W., Bright, J., van der Walt, S. J., Brett, M., Wilson, J., Millman, K. J., Mayorov, N., Nelson, A. R. J., Jones, E., Kern, R., Larson, E., Carey, C. J., Polat, İ., Feng, Y., Moore, E. W., VanderPlas, J., Laxalde, D., Perktold, J., Cimrman, R., Henriksen, I., Quintero, E. A., Harris, C. R., Archibald, A. M., Ribeiro, A. H., Pedregosa, F. and van Mulbregt, P. (2020). *Nature Methods*, **17**(3), 261–272.
URL: *https://www.nature.com/articles/s41592-019-0686-2*

Wright, J. P., (2020). FABLE-3DXRD/ImageD11.
URL: *https://github.com/FABLE-3DXRD/ImageD11*
